# The tumor microenvironment: adding pieces to the puzzle

**DOI:** 10.3389/fimmu.2025.1731338

**Published:** 2026-01-12

**Authors:** Dolores Aguilar-Cazares, Mario Perez-Medina, Jesus J. Benito-Lopez, Miriam Galicia-Velasco, Manuel Meneses-Flores, Angel Camarena, Jose S. Lopez-Gonzalez

**Affiliations:** 1 Laboratorio de Investigacion en Cancer Pulmonar, Departamento de Enfermedades Cronico-Degenerativas, Instituto Nacional de Enfermedades Respiratorias “Ismael Cosio Villegas”, Mexico City, Mexico; 2 Department of Research, Asociación Para Evitar la Ceguera en México IAP, Mexico City, Mexico; 3 Laboratorio de Inmunobiologia y Genetica, Instituto Nacional de Enfermedades Respiratorias “Ismael Cosío Villegas”, Mexico City, Mexico

**Keywords:** extracellular vesicles, immune checkpoints, immune response, metabolic reprograming, resistance, stroma cells, tissue remodeling, tumor evasion mechanisms

## Abstract

In the tumor microenvironment, malignant cells coexist and interact with each other and with stromal, immune, and endothelial cells, as well as with extracellular matrix proteins. The interaction occurs through membrane contact or the production of multiple soluble factors. The composition of tumor and matrix cells changes continuously during tumor development, along with the infiltration of immune cells, forming heterogeneous niches that vary in space and time. We integrate current knowledge about the complex interaction between heterogeneous cell populations in the TME and the impact of these networks in supporting immune defense, which paradoxically promotes tumor progression. We summarize the involvement of immune cells and highlight the impact of certain homeostatic processes mediated by stromal cell populations and matrix components on tumor development. We propose the role of metabolic reprogramming and oxidative stress, as well as extracellular vesicle-mediated signaling, in conferring tumor resistance and therapeutic strategies to disrupt pro-tumor communication networks while enhancing anti-tumor immunity. Our goal is to provide a comprehensive framework for understanding and addressing the cellular interactions underlying cancer progression, fostering opportunities to formulate strategies that control tumor growth and eliminate resistance to treatment options. This integrative perspective provides a basis for designing multi-targeted immunotherapies aimed at rewiring pro-tumor communication networks.

## 1 Introduction

The tumor microenvironment (TME) is a dynamic and heterogeneous ecosystem in which malignant cells interact directly or indirectly with stromal, immune, endothelial cells, and proteins of extracellular matrix (ECM). Far from being a passive scaffold, the TME actively shapes tumor initiation, progression, immune evasion, and therapeutic resistance through an intricate network of intercellular communications and molecular signaling.

Over the past two decades, high-dimensional profiling techniques, including single-cell transcriptomics, proteomics, and spatially resolved mapping, have provided unprecedented insights into the cellular and molecular complexity of the TME. These studies have revealed that cell–cell communication within tumors involves soluble mediators such as cytokines, chemokines, and growth factors (ligands) that bind to plasma membrane-bound receptors, in addition to extracellular vesicles, and direct membrane-membrane physical contacts (1–3). Collectively, these exchanges regulate the crosstalk among stromal, immune, and cancer cells themselves.

A defining feature of TME is its dual capacity to modulate the immune response. While specific subsets of immune cells retain cytotoxic functions, others acquire regulatory or exhausted phenotypes that suppress immunity and facilitate tumor growth. Stromal components, including cancer-associated fibroblasts (CAFs), tumor-associated macrophages (TAMs), and endothelial cells, further contribute to this shift by secreting pro-tumor mediators, promoting cell fusions, remodeling the ECM architecture, stimulating angiogenesis, and supporting metastatic dissemination.

This review synthesizes and integrates current insights into the complex interactions among heterogeneous cell populations in the tumor microenvironment, with a particular focus on how these networks support immune defense yet paradoxically promote tumor progression. We examine the roles of key immune and stromal cell populations, the impact of metabolic and oxidative stress, and the role of extracellular vesicle-mediated signaling. Additionally, we emphasize emerging therapeutic strategies that aim to disrupt pro-tumoral communication networks while enhancing antitumor immunity. By integrating recent findings, we seek to provide a comprehensive framework for understanding and targeting the cellular crosstalk that underlies cancer progression and therapeutic resistance.

## 2 Relevance of inflammation and immune surveillance in the recognition and elimination of emerging transformed cells

Within the TME, a complex and constantly evolving network of interactions occurs among malignant cells, non-malignant stromal and immune cells, and the acellular compounds of ECM. In healthy tissues, exposure to physical, chemical, or biological genotoxic agents continuously induce genomic and epigenomic alterations. Such alterations lead to DNA damage and genomic instability, disrupting key cellular functions. These include sustained proliferative signaling, loss of cell death mechanisms, and changes in genes encoding molecules essential for cell death induction, among others. In the premalignant phase, genomic and epigenetic changes generate structural alterations in proteins (neoantigens) or induce overexpression of self-proteins. Natural killer (NK) cells, innate lymphoid cells specialized in immune surveillance, are critical for detecting and eliminating such aberrant cells (4).

The cytotoxic–regulatory balance of NK cells, classically defined by inhibitory interactions with major histocompatibility complex (MHC) class I molecules and activating signals through stress ligands such as MICA/B, acquires new meaning within the tumor context (5). Stromal- and tumor-derived cytokines remodel this equilibrium, progressively converting immune surveillance into tolerance. Even the perforin–granzyme axis, once the hallmark of NK-mediated cytotoxicity, becomes blunted or repurposed under chronic exposure to transforming growth factor-β (TGF-β), interleukin-10 (IL-10), and hypoxic stress (6). Rather than a fixed killing program, the NK–tumor interface acts as a dynamic rheostat integrating metabolic cues and cytokine availability (7).

NK cell activity is amplified by invariant natural killer T (iNKT) cells, which express a semi-invariant T cell receptor (TCR) recognizing glycolipid antigens presented by CD1d molecules on transformed or stressed cells. Upon antigen recognition, iNKT cells release cytokines such as interferon-γ (IFN-γ), which stimulates NK proliferation and cytotoxicity, establishing a positive feedback loop that strengthens innate immune surveillance. Given the assumption that the human body is in constant exposure to mutagenic insults, the NK–iNKT axis plays a central role in early tumor immunosurveillance.

In injured and dead cells, intracellular molecules are released or translocated to the plasma membrane, serving as alarm signals for immune recruitment. These damage-associated molecular patterns (DAMPs) and, in cancer, lifestyle-associated molecular patterns (LAMPs) (8) or xenobiotic-associated molecular patterns (XAMPs) (9), act as “find me” and “eat me” cues recognized by pattern recognition receptors (PRRs) on phagocytes such as macrophages and dendritic cells. PRRs include membrane-bound Toll-like receptors (TLRs) and C-type lectin receptors (CLRs), as well as cytosolic sensors such as RIG-I-like receptors (RLRs), AIM2-like receptors (ALRs), cyclic GMP–AMP synthase (cGAS), and NOD-like receptors (NLRs) (10). These systems enable rapid detection of cellular stress and initiate both innate and adaptive immune responses (9, 11).

Activation of PRRs on tissue-resident macrophages induces the secretion of growth factors, chemokines (CXCL4, CXCL8, CXCL10, CXCL12, etc.) and cytokines, including CCL2/MCP-1, CXCL13, IL-1α, IL-1β, IL-6, IL-8, IL-12, IL-23, and tumor necrosis factor-α (TNF-α). DAMPS and chemokines that reach the endothelium induce the expression of an array of adhesion molecules to promote the recruitment, rolling, adhesion, and diapedesis of neutrophils. Neutrophils and phagocytes at the site of injury generate reactive oxygen species (ROS) via NADPH oxidase and reactive nitrogen species (RNS) through inducible nitric oxide synthase (iNOS), inflicting oxidative and nitrosative damage on lipids, proteins, and DNA of early transformed cells (12).

For complete tumor eradication, adaptive immunity must be engaged. DCs, the most potent antigen-presenting cells (APCs), phagocytose tumor material, process antigens, and migrate to lymph nodes, where they present peptides via MHC class I and II molecules to naïve CD8^+^ and CD4^+^ T cells, respectively (13). Antigen recognition through the TCR–MHC interaction delivers the first activation signal, costimulatory interactions such as CD80/86–CD28 provide the second, and cytokines like IL-12 or IFN-α supply the third, directing T cell differentiation.

During the activation of the immune response, innate cells and the clonal expansion of CD4^+^ and CD8^+^ T lymphocytes require a large amount of available bioenergy products. Glucose consumption and the synthesis of macromolecules such as nucleotides, lipids, and proteins are necessary for their optimal functioning (14). This process, known as the Warburg effect, is similar to that described in malignant cells, which switch to aerobic glycolysis while maintaining fatty acid oxidation and amino acid uptake to meet biosynthetic demands. This metabolic reprogramming has recently been proposed as a fourth signal (15–18). After activation of CD4^+^ T cells, they differentiate into Th1 cells producing IL-2 and IFN-γ, whereas CD8^+^ cytotoxic T lymphocytes (CTLs) synthesize granzyme and perforin to eliminate tumor cells presenting cognate antigens (19–21).

DAMPs, LAMPs chemokines, and cytokines act as a secondary communication layer, translating immune-mediated injury into stromal activation. Endothelial and fibroblast responses, vasodilation, adhesion molecule expression, and matrix remodeling, facilitate immune infiltration but also seed the architecture of future tumor niches. Proteases, reactive oxygen and nitrogen species, while initially cytotoxic, simultaneously erode extracellular matrix integrity and generate bioactive fragments that signal repair (22). As inflammation resolves, neutrophils and macrophages transition toward pro-resolving phenotypes, initiating tissue regeneration yet preserving the molecular memory of stress that can later be co-opted for tumor growth (23).

## 3 Events related to tissue remodeling create favorable local conditions for the development of cancer

When the coordinated activity of innate and adaptive immune cells achieves complete elimination of incipient transformed tumor cells, the acute inflammatory phase subsides. At this stage, a network of regulatory mechanisms, mediated by soluble factors and cellular interactions, actively limits inflammation and initiates tissue repair, restoring homeostasis (24).

As mentioned above, different immune cells consume nutrients to perform their effector function, releasing waste compounds. Interestingly, these waste products can modulate the immune response, reduce local inflammation and maintain immune homeostasis for successful tissue remodeling. A number of waste products act as immunomodulators, promoting events ranging from the inhibition of the cytotoxic capacity of NK cells and CD8^+^ effector T cells, to the alteration of immune cell differentiation patterns, to the promotion of regulatory T cells (Tregs), among others (25). At high concentrations of lactate or other local waste components, these Tregs are generated from naive, memory, or tissue-resident T cells. They act through two processes: one mediated by cell–cell contact, through the expression of molecules designed as immune checkpoints, or through the release of soluble factors with inhibitory activity.

Neutrophils are key initiators of the resolution phase. Beyond their pro-inflammatory functions, they produce specialized pro-resolving mediators (SPMs) including resolvins and lipoxins, a superfamily of endogenous bioactive lipids that play central roles in this transition regulating inflammation and restore tissue homeostasis (26). During resolution, neutrophils undergo apoptosis, releasing enzyme-rich microparticles that contribute to SPM biosynthesis (27).

SPMs, derived from omega-3 and omega-6 polyunsaturated fatty acids (PUFAs) through the enzymatic activity of lipoxygenases (LOX) and cyclooxygenases (COX), include lipoxins, resolvins, protectins, and maresins. Unlike classical pro-inflammatory eicosanoids (e.g., prostaglandins and leukotrienes), SPMs actively terminate inflammation and promote tissue repair without causing immunosuppression (28). Lipoxins, generated from arachidonic acid (AA; C20:4, ω-6) via transcellular LOX pathway, were the first pro-resolving mediators identified. LXA_4_ and LXB_4_ counteract excessive inflammation and signal the onset of resolution (29). Resolvins, named for their role in the “resolution phase,” are subdivided into E-series (EPA-derived) and D-series (DHA-derived), which include RvE1, RvE2, RvD1, and RvD2. Protectins (PD), also known as neuroprotectins in neural tissues, are DHA-derived and attenuate inflammatory damage while promoting cell survival. Maresins (MaR), primarily synthesized by macrophages from DHA, facilitate clearance of death cells and stimulate tissue regeneration; examples include MaR1 and MaR2. Additionally, cysteine-conjugated SPMs (cys-SPMs), such as maresin conjugates in tissue regeneration (MCTRs) and protectin conjugates in tissue regeneration (PCTRs), arise from glutathione-dependent pathways. Together, these lipid mediators form a coordinated network of “immunoresolvents” that exert potent effects at picomolar to nanomolar concentrations (29).

In addition, neutrophil–platelet interactions also promote lipoxin formation: neutrophils supply leukotriene A_4_ (LTA_4_) via the 5-LOX pathway, which platelets convert to LXA_4_ through 12-LOX activity. This process exemplifies the “lipid mediator class switch,” whereby pro-inflammatory mediators give way to pro-resolving mediators (30). Other proteins, such as annexin A1, galectin-1, and chimerin, secreted by fibroblasts and endothelial cells cooperate to halt leukocyte infiltration, counter-regulate pro-inflammatory signals, and enhance efferocytosis of apoptotic neutrophils (31).

SPMs also orchestrate macrophage polarization. Classically activated M1 macrophages, which produce pro-inflammatory eicosanoids and cytokines (IL-1β, TNF-α, IL-6, IL-8, IL-23), gradually shift to a resolving M2 phenotype under the influence of SPMs. M2 macrophages secrete anti-inflammatory mediators such as IL-10, PDGF, and TGF-β, and they actively produce maresins, resolvin D, and protectins (32, 33). This shift is tightly regulated by metabolic reprogramming in the microenvironment, characterized by increased glutamine utilization, reduced glucose availability, acidic pH, and hypoxia, which favors oxidative phosphorylation over glycolysis and enhances efferocytosis (34).

Anti-inflammatory cytokines from M2 macrophages (IL-4, IL-10, IL-6, TGF-β) stimulate fibroblasts to produce proangiogenic and ECM components, including VEGF, FGF, PDGFD, fibronectin, hyaluronic acid, proteoglycans, and collagen, critical for re-epithelialization. Fibroblasts may also transdifferentiate into contractile myofibroblasts, which contribute to wound closure through production of matrix metalloproteinases (MMPs) and structural ECM proteins (35, 36). This remodeling restores tensile strength and tissue integrity.

**Figure 1:**
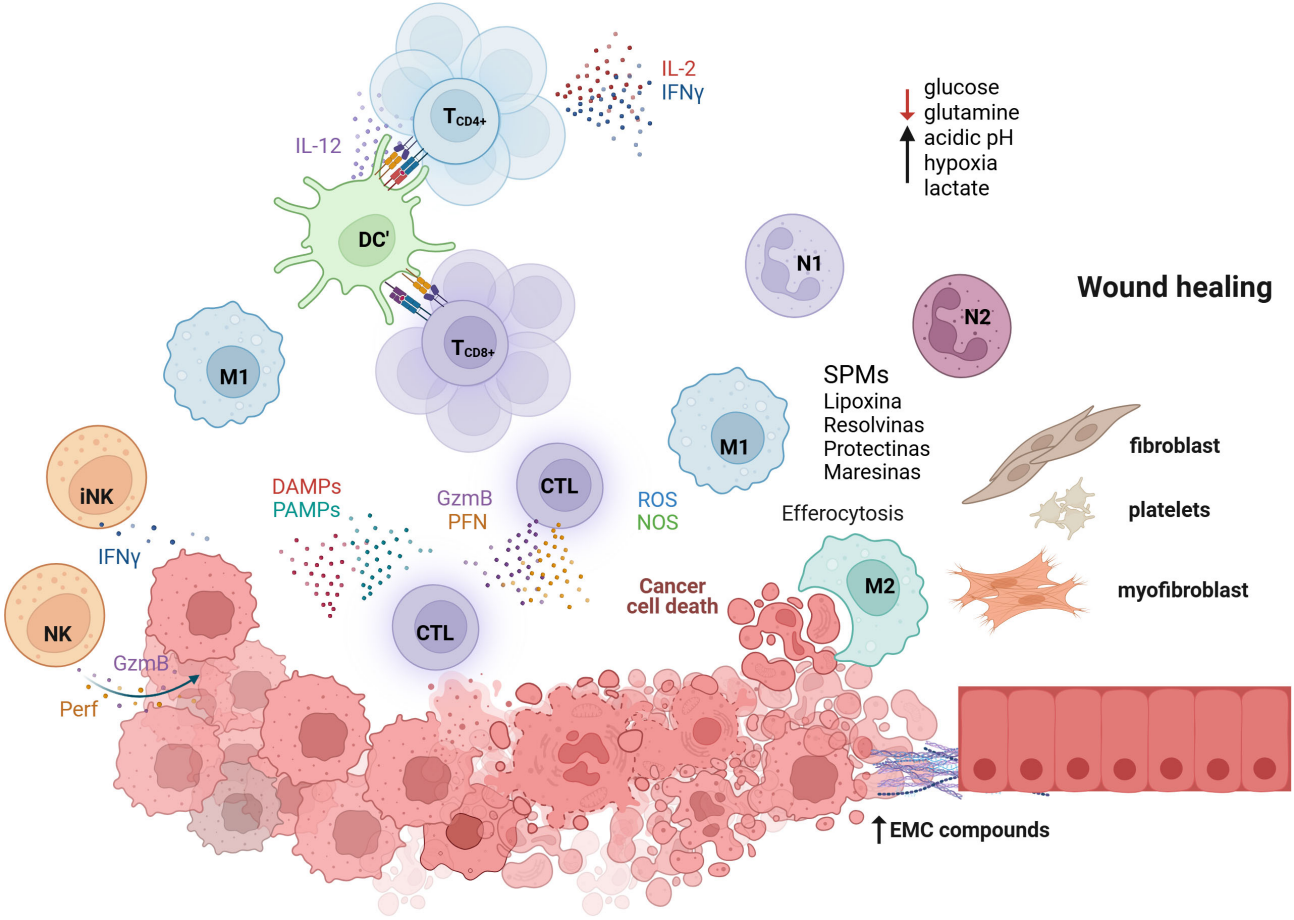
Network of cellular and molecular events for tissue regeneration. Innate and adaptive immune cells cooperate to eliminate transformed cells through cytotoxic and inflammatory mechanisms. Natural killer (NK) and invariant NK (iNK) cells release perforin (PFN) and granzyme B (GzmB) to induce tumor cell death, while dendritic cells (DCs) activate CD4^+^ and CD8^+^ T cells via IL-12 signaling, leading to IL-2 and IFN-γ secretion that amplifies cytotoxic activity. Damage- and pathogen-associated molecular patterns (DAMPs and PAMPs) recruit neutrophils (N1) and M1 macrophages, which generate reactive oxygen and nitrogen species (ROS, NOS) to promote further tumor clearance. As dying cells accumulate, macrophages initiate efferocytosis and metabolic reprogramming under a microenvironment characterized by low glucose and glutamine availability, acidic pH, hypoxia, and increased lactate. This shift favors the production of specialized pro-resolving mediators (SPMs) such as lipoxins, resolvins, protectins, and maresins, and the secretion of anti-inflammatory cytokines (IL-10, TGF-β) and growth factors (VEGF, FGF). These mediators orchestrate fibroblast activation, myofibroblast differentiation, and extracellular matrix (ECM) deposition, leading to re-epithelialization and tissue repair, processes that, while essential for resolution of inflammation, may also set the stage for a regenerative or pre-neoplastic niche. Color and arrows: Black arrows indicate activation or production; red arrows indicate inhibition or depletion.

Collectively, changes in the cellular processes of immune cells, induced by Tregs, other immunomodulatory cells like M2, or high concentration of metabolic by-products, are critical mechanisms for regulating the activity of immune cells (37). In summary, coordinated crosstalk among immune cells, stromal populations, and resident tissue cells not only ensures the elimination of transformed cells but also establishes a tightly controlled resolution phase. The *in situ* biosynthesis of SPMs limits inflammation locally, prevents chronic inflammatory states, and promotes tissue regeneration through synchronized and highly regulated repair mechanisms that preserve the structural and functional integrity of the affected tissue (See **Figure 1**). These complex and redundant mechanisms involve the participation of various cells that generate a metabolic change, promoting regulatory functions in immune cells through the release of soluble factors with antiproliferative activity or the expression of a series of molecules to suppress immune activity. In addition, cellular restructuring occurs through the induction of changes in cytoskeletal adhesion molecules. Fibroblasts and transdifferentiated myofibroblasts involved in the deposition of extracellular matrix components for tissue repair must develop cytoprotective mechanisms to prevent cell death, all in an environment of factors that promote endothelial activity, initiating the process of angiogenesis (38). This physiological process can trigger a pathophysiological environment when these mechanisms are dysregulated by the persistence of some residual transformed cells, creating persistent oxidative stress that induces greater genomic instability and generates microenvironmental conditions that favor the development of cancer.

## 4 From residual cells to cancer promotion: genomic alterations and metabolic reprogramming

The host immune response may fail to eradicate some clones of nascent tumor cells. In such scenarios, oxidative stress generated by macrophages and neutrophils plays a dual role. While they can destroy transformed cells, they may also induce further genomic alterations in the resistant tumor subsets.

Recently, genomic instability can also arise through cell–cell fusion. Hybrid cells represent a pathophysiological process promoted by certain molecules acting as fusogens that is classified as homotypic when cancer cells with slight genetic differences between them fuse, increasing intratumoral heterogeneity; whereas heterotypic fusion between tumor and normal cells generates hybrids with mixed phenotypes (39–41). During early stages of tumor development, when inflammation and cytokines predominates, some signaling pathways appear to be involved in inducing fusion between tumor cells and immune cells like tissue macrophages has been documented (42).

The overlapping expression of tumor and non-tumoral tissue markers has been used to identify hybrid tumor cells. Such cells have also been detected in cancer patients following bone marrow transplantation, via donor–recipient allele analysis (42, 43). In any condition, residual tumor cells continue to accumulate genomic and epigenetic alterations over time, some lethal, others promoting tumor growth or resistance to immune-mediated cytolysis.

Progressive and stochastic genomic instability produces diverse tumor cell clones with specific metabolic adaptations, collectively forming a heterogeneous tumor mass. Sustained tumor expansion, driven by intrinsic mutations and potentially by the formation of homotypic or heterotypic hybrids, imposes high nutrient demands for macromolecule synthesis. In this aspect, tumor cells reprogram their metabolism state toward anabolic pathways that maintains the demand for energy and metabolites necessary for their continuous proliferation rate, increasing aerobic glycolysis and glutaminolysis for production of cellular building blocks needed to generate novel biomass (44). This metabolic switch results in high lactate production, extracellular acidification linked to hypoxia, and alterations in amino acid, creatine, and lipid metabolism (45–47). Hypoxia, in addition to inflammation, is considered to be another trigger for cell-cell fusion events, and the resulting hybrid cells have been associated with autophagy and increased survival in hostile environments (48–50).

**Figure 2:**
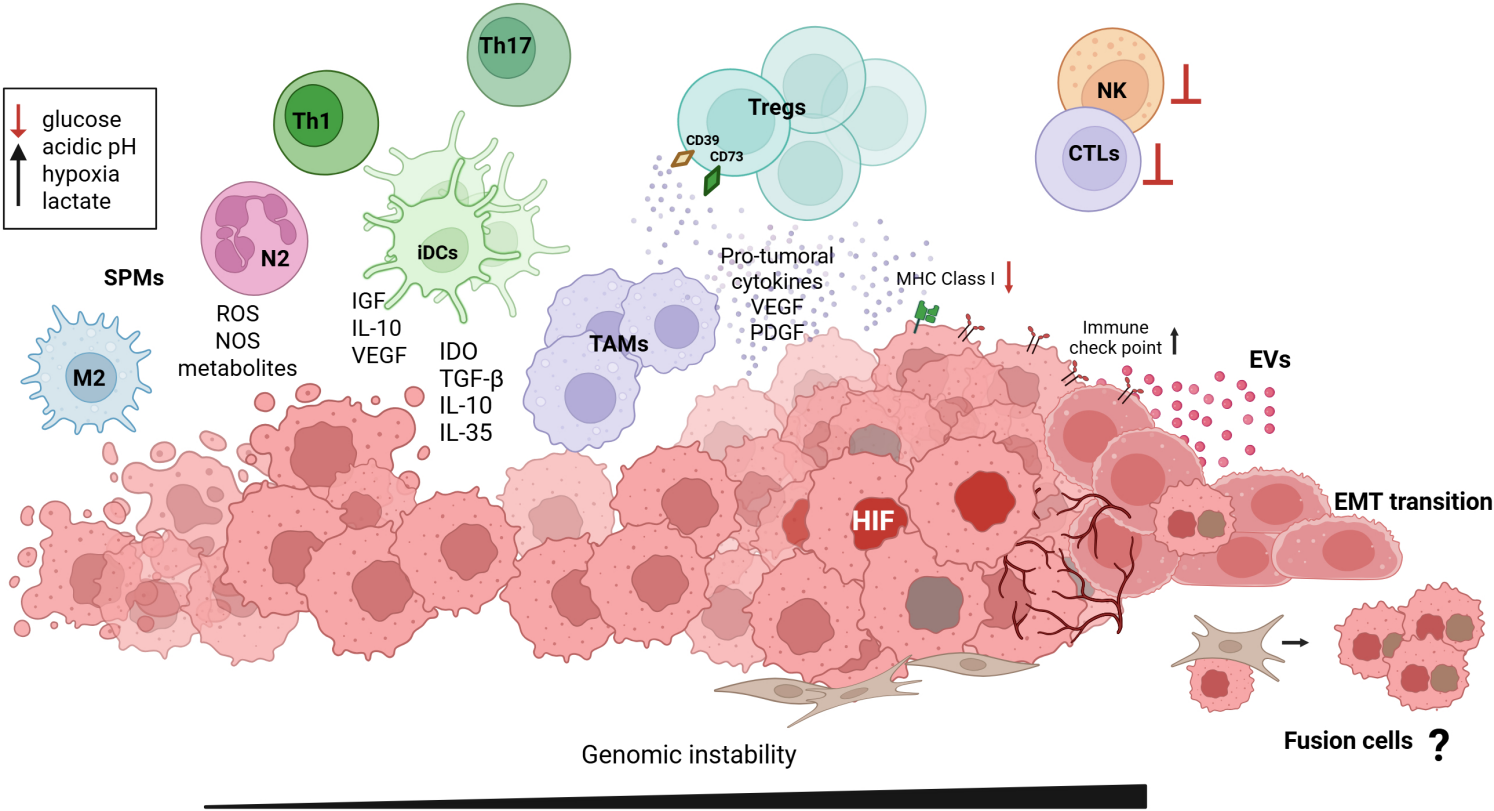
Network of cellular and molecules interactions promoting cancer development. Following tissue re-epithelialization, soluble mediators released by stromal and immune cells, drive progressive adaptation to environmental stress. This shift toward aerobic glycolysis (Warburg metabolism) sustains proliferation, alters apoptotic programs, and promotes the emergence of multiple hallmarks of cancer. Metabolic byproducts, cytokines, and extracellular vesicles (EVs) secreted by tumor cells reprogram the surrounding immune and stromal compartments, shaping a progressively immunosuppressive and pro-angiogenic niche. Within this metabolically constrained microenvironment, characterized by low glucose, acidic pH, hypoxia, and elevated lactate, cytotoxic T lymphocyte (CTL) and NK cell activity are inhibited (⊥). Regulatory T cells (Tregs), tolerogenic dendritic cells (iDCs), and tumor-associated macrophages (TAMs) produce IL-10, TGF-β, IDO, IL-35, VEGF, and PDGF, which suppress effector immunity and promote vascular remodeling. Th1/Th17 imbalance, together with N2 neutrophils and M2 macrophages, reinforces immune evasion through specialized pro-resolving mediators (SPMs) and oxidative metabolites. At the tumor front, HIF stabilization, EV signaling, and loss of MHC class I expression cooperate with immune checkpoint upregulation (PD-L1, CTLA-4) to blunt antitumor responses. The activation of EMT programs and stromal–tumor crosstalk facilitates fibroblast activation, cell fusion, and acquisition of hybrid phenotypes, promoting invasion, metastasis, and persistence of drug-tolerant persister (DTP) cells within an ecosystem of expanding genomic instability. Color and arrows: Black arrows indicate activation or production; red arrows indicate inhibition or depletion.

Importantly, these metabolic shifts not only reflect adaptation to environmental stress but also drive clonal selection under immune pressure, favoring the expansion of immune-evasive phenotypes (51). The various metabolites produced by this environment not only influence the activity of multiple immune cells, but also affect stromal cells located within the tumor itself or far from its growth (See **Figure 2**).

## 5 Tumor microenvironment and major signaling pathways

Ongoing tumor growth perpetuates genomic instability and cellular heterogeneity. Tumors develop in close association with stroma composed of inflammatory and immune cells, endothelial cells, pericytes, cancer-associated fibroblasts (CAFs), and the ECM. Tumor niches can arise from the gain of intrinsic mutations in tumor cells, from fusion between genotypically distinct tumor cells, between tumor cells and different immune cells, or from interactions with stromal cells, which encourages excessive tumor cell growth and cellular heterogeneity (52).

In any event, within these niches, continuous intercellular communication occurs through soluble mediators binding to specific receptors, triggering autocrine, paracrine, or juxtacrine signaling. These interactions promote tumor growth and progression, amplified by increased synthesis of growth factors, cytokines, and chemokines, as well as overexpression or enhanced affinity of membrane receptors.

Below is a brief summary of some of the key signaling pathways that are frequently overexpressed in cancer and their biologic effects.

### 5.1 JAK/STAT signaling pathway

The Janus kinase–signal transducer and activator of transcription (JAK–STAT) pathway is activated when proinflammatory cytokines or growth factors bind to transmembrane receptors, leading to JAK recruitment and phosphorylation. Activated JAKs phosphorylate STAT proteins, which dimerize and translocate to the nucleus, regulating genes involved in proliferation, differentiation, inflammation, immune activation, and apoptosis (53).

Aberrant activation, via JAK/STAT overexpression or loss of negative regulators such as PTPs, PIAS, or SOCS, promotes tumorigenesis by enhancing proliferation, matrix metalloproteinase (MMP)-mediated invasion, epithelial–mesenchymal transition (EMT), metastasis, and resistance to cell death.

### 5.2 PI3K/AKT/mTOR signaling pathway

Activated by receptor tyrosine kinases, integrins, G protein–coupled receptors, RAS, or lipophilic hormones, PI3K phosphorylates membrane inositol phospholipids to generate second messengers that recruit and activate Akt (protein kinase B) and mTOR. In parallel, RAS, normally inactive in its GDP-bound form, undergoes GTP loading upon stimulation, initiating multiple effector cascades, including MEKK/SEK/JNK, Raf/MEK/ERK, and PI3K/Akt/NF-κB pathways (54). Together, these interconnected modules orchestrate transcriptional programs controlling cell cycle progression, survival, metabolism, and motility. Dysregulation of these axes confers proliferative, anti-apoptotic, and invasive advantages that underpin the hallmarks of cancer (55).

### 5.3 Cadherin–catenin/WNT pathways

The cadherin–catenin complex maintains epithelial integrity via calcium-dependent cell–cell adhesion. Loss of cadherin disrupts adherents junctions, releasing β-catenin, which is degraded unless WNT signaling inhibits its phosphorylation (56). Stabilized β-catenin translocates to the nucleus, activating LEF/TCF target genes. WNT signaling regulates stem cell renewal, proliferation, differentiation, and motility (57). Loss of E-cadherin promotes EMT, tumor progression, metastasis, and poor prognosis. Oncogenic pathways such as MAPK, Ras, Rac1, PI3K/AKT, and TGF-β can disrupt cadherin–catenin adhesion.

In addition, gap junctions and tunneling nanotubes (TNTs) enable direct exchange of ions, metabolites, proteins, RNA, and organelles, supporting chemoresistance, metabolic coupling, and immune evasion (58, 59).

### 5.4 TGF-β signaling pathway

TGF-β is secreted in a latent form bound to latency-associated peptide (LAP) and stored in the ECM. It is activated by ROS, integrins, proteases, and thrombospondin-1 (60, 61). Active TGF-β binds TGF-βRII, recruiting TGF-βRI and initiating SMAD-dependent transcriptional regulation. Noncanonical signaling involves MAPK, PI3K/AKT/mTOR, and JNK pathways. TGF-β is essential for tissue repair, fibroblast activation, ECM production, angiogenesis, and myofibroblast differentiation. However, in cancer, it promotes EMT, immune suppression, metastasis, and therapy resistance (62, 63).

In summary, these important signaling pathways and others not included in this review due to space limitations form interconnected feedback networks within the TME. By integrating extracellular cues into transcriptional programs, they shape tumor cell behavior and contribute to immune evasion, progression, and therapeutic resistance.

## 6 Complex interactions in the tumor microenvironment and their impact on tumor immune evasion

Tumor development and progression are also driven by the intricate communication networks that regulate metabolic fluxes, immune responses, and tissue remodeling. Multiple models highlight the pivotal role of intercellular communication in shaping the process of immunoediting and facilitating the transition to immune escape.

The immunoediting concept posits that immune responses continuously sculpt the tumor cell phenotype. Under immune pressure, sensitive tumor cells are eliminated, while those capable of resisting destruction or evading detection survive and expand (64). This selection process is strongly influenced by the spatial and temporal dynamics of intercellular signaling with dysregulated growth factors and cytokine production and tumor cell metabolic reprogramming. Recently, the Galluzzi group has integrated into the “three Cs” model the mechanisms that tumor cells develop to evade immune recognition, proposing events such as camouflage, coercion, and cytoprotection as distinctive hallmarks of cancer immune evasion (65).

Overproduction of growth factors and cytokines, or gain-of-function mutations in membrane receptors or intracellular trafficking molecules, can sustain the uncontrolled proliferation of tumor cells. These events may also compromise immune function by limiting the availability of soluble mediators at concentrations sufficient to support robust anti-tumor responses. Furthermore, the high proliferative rate of tumor cells depletes essential nutrients and increases metabolic by-products, predominantly through aerobic glycolysis, to sustain their growth.

Metabolic by-products such as ROS, lactate, and oncometabolites function as signaling mediators that modulate immune cell activation, polarization, and survival, thereby contributing to immunosuppression. Within the TME, gradients of lactate, glutamine, acidity, and hypoxia create spatially distinct niches with unique cellular and noncellular compositions (66, 67). These conditions are largely orchestrated by hypoxia-inducible factor-1 (HIF-1), which regulates key enzymes in glycolysis and lipid metabolism (67–69).

Metabolic reprogramming also profoundly influences phagocyte behavior. During acute inflammation phase, macrophages adopt a pro-inflammatory M1 phenotype, secreting IL-1, TNF-α, IL-6, IL-12, IL-23, and other cytokines that sustain innate immune activity and promote Th1 adaptive responses. Th1-derived IFN-γ reinforces M1 activity. Upon resolution of the insult, macrophages typically transition to an M2 (pro-resolving) phenotype, engaging in efferocytosis of apoptotic cells and debris, and secreting IL-10, TGF-β, and growth factors to coordinate tissue repair. SPMs, such as maresins (MaRs) produced by macrophages, accelerate the M1-to-M2 shift, enhancing debris clearance and fostering an anti-inflammatory milieu (70).

In the TME, however, M1-mediated antitumor activity is progressively suppressed, facilitating the predominance of M2-like TAMs. Sustained tumor growth alters the local environment through persistent release of soluble mediators, CSF-1, G-CSF, IL-6, IL-10, CCL2, CXCL12, PDGF, VEGF, among others, produced by both tumor cells and reparative M2 macrophages (71). These signals promote TAM polarization and differentiation into a hybrid phenotype with features of both M1 and M2 macrophages.

Environmental stressors, hypoxia, acidity, nutrient deprivation, and tumor-derived metabolites, in combination with soluble factor signaling, further drive TAM differentiation. TAMs then secrete growth factors, cytokines, and chemokines that fuel tumor proliferation via autocrine and paracrine loops, or through exosome-mediated communication. This promotes endothelial activation, recruitment of additional monocytes, and maintenance of TAM heterogeneity. In advanced cancer stages, these subsets preferentially infiltrate specific tumor niches (72, 73).

Recent high-resolution profiling techniques, including single-cell RNA sequencing (scRNA-seq), CyTOF, and spatial transcriptomics, have revealed that TAMs exist along a continuous spectrum of transcriptional states, rather than fitting the classical M1/M2 dichotomy. scRNA-seq studies consistently identify functionally distinct TAM clusters, such as MHC-II^high^ antigen-presenting TAMs, MHC-II^low^ immunosuppressive TAMs, and SPP1^+^ TAMs, the latter preferentially enriched in hypoxic niches and associated with angiogenesis, extracellular matrix remodeling, and tumor progression (74).

Within a single tumor, TAMs display remarkable phenotypic diversity and occupy specialized microanatomical regions, reflecting adaptation to gradients of hypoxia, nutrient availability, cytokines, and lipid metabolites. Their high plasticity allows rapid transcriptional and metabolic reprogramming in response to local cues. Intriguingly, emerging evidence suggests that specific TAM subsets may even arise through heterotypic fusion events between macrophages and tumor cells, potentially generating hybrid populations with enhanced protumoral capabilities, an attractive but still evolving hypothesis that warrants further investigation (75).

## 7 Cell metabolism and stroma—immune modulation in the TME

Competition for nutrients and the profound metabolic rewiring of the TME serve as critical determinants of immune suppression. Tumor cells, cancer-associated fibroblasts (CAFs), endothelial cells, tumor-associated macrophages (TAMs), myeloid-derived suppressor cells, and infiltrating lymphocytes coexist in a landscape of restricted glucose, amino acids, oxygen, and lipids. These constraints do not simply reflect metabolic stress; rather, they function as potent regulatory cues that bias immune cells toward exhaustion, tolerance, or protumoral phenotypes (76).

A paradigmatic example is the Warburg effect, where tumor cells preferentially utilize aerobic glycolysis even in oxygen-rich conditions. This shift results in massive glucose consumption and lactate secretion, lowering extracellular pH and generating an environment that promotes M2-like TAM polarization, reduces NK and CD8^+^ T cell cytotoxicity, and impairs dendritic cell (DC) activation (77).

Glutamine dependency is also significant, tumor cells upregulate transporters such as SLC1A5 and GLS, allowing proliferation even in nutrient-poor environments, while limiting glutamine availability for effector T cells (78). Arginine is an essential amino acid for T cell proliferation, survival, and effector function. Tumor cells and TAMs both depend on arginine, but TAMs express high levels of arginase-1, which depletes extracellular arginine and profoundly impairs T cell activation and expansion following antigen presentation by APCs (79–81). Arginine depletion restricts mitochondrial respiration and blocks AKT/mTOR activation, reinforcing T cell exhaustion in the TME (82).

Tryptophan metabolism constitutes another crucial immunoregulatory pathway. Mature DCs, macrophages, endothelial cells, and specific epithelial cells upregulate the indoleamine-2,3-dioxygenase (IDO) in response to inflammatory stimuli such as LPS, Toll-like receptor ligands, CpG DNA, IL-1β, TNF-α, IL-6, and IFN-α/β. Physiologically, IDO limits tissue damage. It preserves homeostasis during tissue repair by catalyzing the degradation of tryptophan (Trp) into immunomodulatory kynurenines (Kyn) and downstream metabolites, including nicotinamide adenine dinucleotide (NAD^+^), an essential cofactor for glycolysis and oxidative phosphorylation.

However, tumors constitutively express IDO and tryptophan-2,3-dioxygenase (TDO/TDO2) accelerating Trp catabolism and amplifying immune suppression (83, 84). Trp depletion activates the GCN2/eIF2α pathway, which in turn inhibits AKT/mTOR signaling, inhibiting T cell proliferation and promoting apoptosis. Meanwhile, Kyn accumulation promotes nuclear translocation of the aryl hydrocarbon receptor (AhR), which complexes with HIF-1β to induce expression of IL-10, IL-17, and IL-22, reinforcing immunosuppression (83, 84). AhR/HIF-1β complexes also upregulate the ectonucleotidases CD39 and CD73, which hydrolyze extracellular ATP (a pro-inflammatory DAMP) into adenosine that promotes immunosuppression. Adenosine not only attenuates cytotoxicity but also promotes angiogenesis and impairs leukocyte extravasation (85). This adenosinergic loop is further reinforced under hypoxia via stabilization of HIF-1α, which enhances CD39/CD73 expression (86).

As was previously mentioned, TGF-β1 represents a master regulator of tissue repair and immune tolerance and is one of the most potent immunosuppressive cytokines in the TME. It is secreted by tumor cells, fibroblasts, and TAMs, and stored as latent form (LTGF-β) in the ECM. Activation involves proteases such as plasmin and thrombospondin-1, matrix metalloproteinases (MMPs), glycoprotein A repetitions predominant protein (GARP), or integrins, and other cofactors. Tregs cells also present membrane-bound LTGF-β, which upon receptor binding triggers SMAD-dependent transcriptional programs (87).

In naïve CD4^+^ T cells, TGF-β induces the transcription factor FOXP3, promoting differentiation into Tregs (88, 89). Recent evidence shows that Treg cells exist as a broad spectrum of heterogeneous subsets, identified by distinct phenotypic markers. In addition, the markers described are nonexclusive of Tregs, as some of them can be found in some populations of effector Th cells. The wide phenotypic heterogeneity is mainly based on their functional capacity to inhibit the immune response, more than specific CD markers, cytokines production, chemokines, transcription factors expression, tissue localization, and homeostatic or pathologic process (88, 89).

Asimnasab-Sorkhabi et al. suggest that Treg cells that share expression of two master transcription factors with other T helper subsets, like Th1 (Tbet+), Th2 (GATA-3+), Th17 (ROR-γt+), and Tfh (Bcl6+) should be named “hybrid Treg cells”. This novel proposal is based on the protumor and antitumor activities demonstrated by these subsets of Tregs (90). Whether coexpression of markers and functionality of the varied Tregs rely on distinct stages of their development promoted by the wide plasticity of T cells previously demonstrated, or this proposal may derive from membrane-membrane cell fusions among Treg cells and the varied phenotypes of T effector cells, event promoted by the tissue environment, requires a deeper investigation, as well as defined the role that the diverse subsets of Tregs plays in TME. **Table 1** summarizes the major nutrient-dependent pathways in the TME, the cellular sources involved, and their functional impact on innate and adaptive immune responses.

**Table 1:** Major immunometabolic pathways in the tumor microenvironment and their impact on antitumor immunity.

*Metabolic mechanism/pathway*	Main source in the TME	Impact on immune cells	Consequence for antitumor immunity	Key evidence/examples	References
Warburg effect (aerobic glycolysis)	Tumor cells, CAFs	Reduces CD8^+^ and NK cytotoxicity; impairs DC maturation	Global immune suppression; expansion of hypoxic niches	↑ LDHA, ↑ GLUT1; exhausted TILs in acidic pH	(76).
Lactate-driven acidification	Tumor cells, TAMs	Increases Tregs; drives M2 polarization; inhibits NK cells	Promotes immunosuppression, angiogenesis, EMT	Elevated plasma lactate → poor prognosis	(77).
Glutamine metabolism (glutaminolysis)	Tumor cells, CAFs	Limits glutamine availability for T cells; supports tumor proliferation	Enhanced immune tolerance and therapeutic resistance	SLC1A5/GLS overexpression in solid tumors	(78).
Arginine metabolism{it} {/it} (Arg → Orn; arginase-1)	M2 TAMs	Inhibits T cell expansion; blocks mTOR–AKT signaling	Functional exhaustion of CD8^+^ T cells	High ARG1 correlates with immunosuppressive TME	(80)
Tryptophan catabolism (IDO/TDO → kynurenine)	Tumor cells, DCs, endothelial cells	Activates AhR; induces Tregs; suppresses Th1 responses	Immune escape and resistance to ICIs	High Kyn/Trp ratio predicts therapy failure	(82, 83).
Hypoxia (HIF-1α activation)	Tumor core, CAFs	Reduces T cell infiltration; promotes M2 and Treg phenotypes	Immune-excluded niches; therapy resistance	HIF-1α upregulates CD39/CD73 → adenosine	(85).
Adenosinergic signaling (CD39/CD73 → adenosine)	Tumor cells, Tregs, endothelial cells	Inhibits CD8^+^ and NK cells; enhances Tregs	Deep suppression of cytotoxic responses	A2A receptor activation → potent inhibition	(92)
Lipid metabolism/FAO in Tregs and TAMs	TAMs, CAFs	Stabilizes suppressive phenotypes	Persistence of Tregs and M2 macrophages	CPT1A activity in intratumoral Tregs	(93)
Oncometabolites (2-HG, succinate, fumarate)	Tumor cells	Epigenetic reprogramming of T cells	Sustained T cell dysfunction	IDH1/2 mutations → 2-HG accumulation	(94)

Advances in non-targeted metabolomics have recently unveiled critical regulators of endothelial and tumor metabolism, such as MFSD8, which orchestrate the balance of lipid and amino acid availability in the TME, further influencing immune cell exhaustion (91).

Despite their heterogeneity, all Tregs subsets potently suppress antitumor immunity through secretion of TGF-β, IL-10, and IL-35, supporting tissue homeostasis, and in the TME tumor reinforcing the immunosuppressive tone (95). This regulatory axis promotes macrophage polarization from M1 to M2 phenotypes, induces ectoenzyme expression in macrophages and APCs that increase local adenosine production, and suppresses T cell proliferation and Th1/Th17 differentiation, reducing secretion of effector cytokines such as IFN-γ, IL-17, and IL-22. These effects extend to cytotoxic lymphocytes, in NK cells and CD8^+^ T cells, TGF-β signaling downregulates granzyme and perforin synthesis, impairing cytolytic activity (94). Collectively, nutrient competition (glucose, glutamine, arginine, methionine, tryptophan), secretion of suppressive metabolites (lactate, Kyn, adenosine), cytokine signaling (TGF-β, IL-10), and metabolic symbiosis between tumor cells and TAMs create a resilient immunosuppressive niche (96, 97). These intertwined networks blunt both innate and adaptive immunity, establishing an immune-privileged environment that supports tumor progression (See **Figure 2**).

## 8 Immune infiltration patterns, tumor immunophenotypes, and stromal contributions

The distribution and density of immune cells within tumor nests and stromal compartments is a defining feature of tumor immunophenotypes. Most cancers are heavily infiltrated by M2-polarized macrophages, accompanied by variable proportions of polyclonal and phenotypically distinct CD3^+^ T cell subsets, including memory CD4^+^/CD8^+^ T cells, Tregs, and follicular helper T cells (98, 99). In contrast, CD56^+^ or CD57^+^ NK cells appear at variable-to-low frequencies, while neutrophils and mast cells are generally scarce (100–102). The composition of tumor-infiltrating lymphocytes (TILs) varies considerably even among tumors of the same histological type (103, 104).

Based on immune cell infiltration, tumors can be broadly categorized into three phenotypes. Immunologically inflamed, or “hot” tumors, exhibit abundant immune infiltrates often associated with highly immunogenic antigens, robust and chronic immune reactivity, and better prognosis, as well as improved responses to therapy. However, immune infiltration alone does not ensure therapeutic success, highlighting the importance of characterizing the specific immune subpopulations present. Immunologically excluded tumors lack T cells in direct contact with tumor cells, with immune cells confined to stromal or peritumoral fibroblast zones, patterns that may arise from MHC-I downregulation, impaired chemotactic factor production, or physical stromal barriers. Immunologically desert, or “cold” tumors, display no detectable T cell infiltration, frequently due to MHC-I loss, defects in the antigen presentation mechanisms, or high levels of inhibitory cytokines from tumor cells, fibroblasts, and myofibroblasts.

The cellular and molecular bases of these immunophenotypes, particularly those that mimic wound-healing environments, remain incompletely understood (105). Advances in spatial transcriptomics and high-resolution tumor mapping have improved immune infiltrate characterization, in hepatocellular carcinoma, these technologies have mapped tumor evolution and revealed highly variable genes associated with TME heterogeneity (106–110). The marked intratumoral heterogeneity requires rigorous analysis using multiple sections of the same tumor to obtain a more accurate representation.

Currently, it is recognized that a subset of tumors develops ectopic lymphoid aggregates known as tertiary lymphoid structures (TLS), which resemble secondary lymphoid organs. Mature TLS contains dendritic cells, macrophages, subsets of T and B lymphocytes, fibroblasts, and endothelial cells, forming organized immune hubs capable of initiating potent adaptive antitumor responses. Their functional relevance is particularly evident in tumors treated with immune checkpoint inhibitors (ICIs). However, tryptophan (Trp) metabolites and stromal-derived factors can impair TLS maturation, limiting their immunological potency. Despite these challenges, TLS presence correlates with improved clinical outcomes, especially in patients receiving ICIs (111–114).

Fibroblasts are ubiquitous mesenchymal cells essential for cell differentiation, tissue morphogenesis, and tissue repair, bone marrow progenitors, mesenchymal stem cells, epithelial or endothelial cells, or malignant epithelial cells. Their plasticity allows differentiation into heterogeneous populations, including stromal fibroblasts, activated fibroblasts, myofibroblasts, and cancer-associated fibroblasts (CAFs). CAFs secrete a diverse repertoire of signaling molecules such as TGF-β, bFGF, IL-6, IL-8, PDGF, VEGF, and HGF, as well as ECM-remodeling enzymes including matrix metalloproteinases (MMPs) and tissue inhibitors of metalloproteinases (TIMPs) (115, 116). Through autocrine and paracrine signaling, as well as extracellular vesicle-mediated exchange, CAFs engage in extensive crosstalk with tumor cells, immune populations, and other stromal elements (116, 117). Functionally, they promote tumor progression by stimulating proliferation via FGF, IL-6, and CXCL12. In addition, the secretory proteases MMPs and urokinase-type plasminogen activator (uPA) that cleave key ECM proteins, including collagen, elastin, and fibronectin, thereby facilitating immune cell infiltration, driving EMT through PDGF, HGF, and IL-1, and enhancing migration and metastasis, effects reinforced by tumor-associated macrophage-derived factors in a pro-tumorigenic feedback loop.

Macrophages, when responding to tissue insults, initiate a respiratory burst that generates ROS capable of damaging lipids, proteins, and nucleic acids, while also activating diverse signaling pathways (118). Cells counter ROS through enzymatic and non-enzymatic antioxidant defenses (119). The Nrf2/HO-1 pathway is a central regulator of these cytoprotective mechanisms, mitigating oxidative stress, suppressing inflammation, and preserving tissue integrity. Under oxidative stress, kinases such as ERK, PI3K, AMPK, and PKC mediate dissociation of Nrf2 from its cytoplasmic repressor Keap1, enabling nuclear translocation and binding to antioxidant response elements (AREs; 5′-RTGAYnnnGCR-3′), which drives transcription of antioxidant genes including superoxide dismutase (SOD), glutathione-S-transferase (GST), catalase (CAT), and γ-glutamylcysteine synthase (γGCS) (120, 121).

The free heme group itself can be pro-oxidant, but heme oxygenase-1 (HO-1), a stress-inducible enzyme, catabolizes it into carbon monoxide, ferrous iron, and biliverdin, subsequently converted to bilirubin, all with antioxidant and anti-inflammatory properties (122, 123). Nrf2 recruitment to the ARE within the HO-1 gene amplifies these cytoprotective programs (124). During wound-healing resolution, hypoxia, acidosis, metabolic shifts, and the transition to anti-inflammatory mediators can activate Nrf2/HO-1 in fibroblasts, myofibroblasts, and TAMs, granting resistance to cell death and enabling coordination of inflammation resolution, angiogenesis, and tissue regeneration (125–128).

In the TME, oxidative stress intensifies, imposing selective pressure on malignant and non-malignant cells to adopt antioxidant and tissue-protective strategies. Hybrid cells from fusion events between tumor cells and fibroblasts, myofibroblasts, TAMs, or bone marrow cells (129) may combine phenotypes with or without prior Nrf2/HO-1 activation, potentially allowing transient quiescence or stem-like states that enhance survival under stress. Although this remains speculative, it complements the view that individual tumor cells can autonomously upregulate Nrf2/HO-1 to evade death. Constitutive HO-1 overexpression, reported in multiple cancers, may or may not indicate hybrid-cell origins.

Beyond survival, tumor–stromal cell fusion may aid immune evasion by incorporating self-antigens from autologous cells into tumor membranes, masking neoantigen recognition and reducing immune detection. Tumor cells may further resist NK and cytotoxic T lymphocyte-mediated killing by suppressing granzyme and perforin activity or altering pro-/anti-apoptotic gene balance, processes potentially modulated by Nrf2/HO-1. Resistance is reinforced by MHC loss through allele deletion or genetic alterations and reduced co-stimulatory molecule expression, impairing T cell activation (130).

Quantitative analyses further illustrate the magnitude and functional consequences of these alterations within the TME. TAMs frequently dominate the myeloid infiltrate, representing 30–50% of all tumor-infiltrating immune cells in pancreatic, breast, lung, and glioma tissues (71). In highly fibrotic tumors, TAM density can surpass that of dendritic cells by more than an order of magnitude, reinforcing an immunosuppressive milieu through IL-10, TGF-β, and ARG1 secretion (131). Similarly, Tregs accumulate in stromal-rich or immune-excluded regions, often outnumbering effector CD8^+^ T cells with Treg: Teff ratios ranging from 2:1 to 5:1 (132). This imbalance correlates with impaired cytotoxic function, reduced granzyme B expression, and diminished responsiveness to checkpoint blockade (133).

Spatial metabolite profiling underscores the metabolic pressures that shape immune fate. Lactate concentrations reach 10–20 mM in hypoxic tumor cores—nearly an order of magnitude higher than in adjacent nonmalignant tissues (<2 mM) (134). This sharp metabolic gradient drives intracellular acidification of T cells, inhibits mTOR signaling, and suppresses cytokine production, while simultaneously supporting M2-like polarization of macrophages and promoting Treg stability (135).

The structural remodeling of the ECM further constrains immune access. Tumors commonly exhibit 200–300% increases in collagen fiber density, accompanied by elevated crosslinking mediated by lysyl oxidase (LOX). These changes raise tissue stiffness into the 1–10 kPa range, comparable to fibrotic organs, creating physical corridors that redirect or fully prevent lymphocyte infiltration (136). Dense collagen bundles can reduce T cell migration speed by 50–70%, while disorganized fibrillar networks alter chemokine diffusion, generating “immune deserts” even in tumors with substantial neoantigen burden (137).

Spatial and temporal heterogeneity within the TME critically shapes treatment responses. ECM remodeling by CAFs and TAMs generates irregular stromal densities, heterogeneous collagen fiber alignment, and hypoxic pockets that restrict T cell infiltration and reduce drug penetration (73). These spatial gradients also influence the distribution of immune phenotypes, with immunosuppressive M2 macrophages enriched in hypoxic cores, while cytotoxic T cells tend to localize toward better-perfused peripheral regions (138). Temporally, dynamic shifts in stromal stiffness, angiogenic activity, and ECM turnover alter the accessibility of tumor nests throughout disease progression and under therapeutic pressure. As a result, both spatial compartmentalization and time-dependent ECM remodeling directly impact the efficacy of immunotherapies, metabolic inhibitors, and cytotoxic regimens, contributing to heterogeneous and often unpredictable treatment outcomes (139).

Together, these quantitative features—immune composition imbalances, metabolite gradients, and ECM stiffening—demonstrate how the TME evolves into a compartmentalized ecosystem that systematically restricts antitumor immunity. They provide a measurable framework for understanding why certain tumors remain refractory to immunotherapy despite robust antigenicity.

## 9 Exosomes mediate communication among tumor, stroma and immune cells

Nowadays, it is accepted that all cell types express an evolutionarily conserved communication system though the cell-derived membrane-surrounded vesicles designated extracellular vesicles (EVs) and contribute to a distinct type of cell-cell fusion. EVs carry molecules of distinct nature and transport information to distant sites via body fluids. The EVs are heterogeneous and comprises microvesicles (MVs) and exosomes that are classified with respect to density, subcellular origin, function and bioactive cargo, like a wide-variety of components of cytosolic and cell surface, diverse nuclear proteins, transcripts, and even fragments of DNA (140–143). EVs can be categorized according their size in exosomes which are mainly derived from endosomal compartment (30–100 nm diameter), microvesicles also called ectosomes, they derive from plasma membrane budding (100–1000 nm diameter), and recently the large oncosomes that seem to be specific for transporting oncogenic material, as they have been detected in malignant process (1-10 μm in diameter) (144). The EV cargo can be transferred to normal or malignant cells, inducing genotypic, phenotypic and cellular functional changes. Various groups have produced excellent reviews on this topic (143, 145, 146) that summarize the information regarding the role of different EVs in various immune cells, both activating antitumor actions and promoting their induction or polarization towards immune cells involved in protumor actions that encourage tumor evasion mechanisms and tumor resistance to treatments.

Within the TME, the production and release of MVs by the various types of cells present, in addition to hypoxia and acidic conditions, metabolic stress, oxidative imbalance, and signalization induced by membrane-membrane and soluble interactions, etc., is an additional factor involved in the remodeling of the phenotype of malignant and non-malignant cells (147). These signals reinforce the loss of cell adhesion, reorganization of the cytoskeleton, and increased migration and invasion in cancer cells (148). This miscellaneous environment and stochastic events that induce in the varied cellular components of the TME, stimulate tumor cells, CAFs, and M2 macrophages to produce and secrete matrix metalloproteinases (MMPs) and other proteases. These enzymes degrade basement membrane components and weaken endothelial junctions, facilitating local invasion and vascular escape by cancer cells (149).

Intercellular membrane fusion and exchange of tumor-derived microvesicles with fibroblasts have been proposed to facilitate EMT and promote drug resistance by transferring oncogenic proteins, RNAs, and signaling lipids (150–153).

Platelets, another key stromal component, have long been recognized for their role in shielding tumor cells from mechanical shear forces in circulation and protecting them from immune-mediated lysis (154). Changes in platelet count or function correlate with disease stage and prognosis (155–157). Increasing evidence indicates that platelets can infiltrate tumors by binding collagen through glycoprotein VI (GPVI) and forming integrin-mediated contacts with tumor cells. Upon degranulation, they release potent proangiogenic factors, including vascular endothelial growth factor (VEGF), fibroblast growth factor (FGF), epidermal growth factor (EGF), and platelet-derived growth factor (PDGF), which collectively drive neovascularization and sustain tumor growth. In turn, tumor cells release exosomes enriched with tumor-specific proteins, growth factors, and mRNA, which are taken up by platelets. This bidirectional exchange not only amplifies local tumor growth but also promotes systemic dissemination, immune evasion, and metastatic colonization (158, 159). While the pro-tumoral roles of platelets are well established, some studies suggest they can exert antitumor effects, a duality likely dictated by tumor type and the diverse bioactive cargo they carry (160).

Recent evidence highlights that specific cargo within tumor-derived EVs actively reshapes the immune landscape. For instance, exosomal PD-L1 has emerged as a critical mediator of immune evasion, capable of inhibiting T cell activation and promoting senescence through lipid metabolism reprogramming, effects that are mechanistically distinct from membrane-bound PD-L1 (161). Furthermore, a mutual regulation exists where tumor-derived exosomes induce PD-L1 expression in TAMs, while TAM-derived exosomes can reciprocally enhance tumor cell immune resistance (162).

Additionally, non-coding RNAs such as miR-21 shuttled by exosomes play a dual role in remodeling the TME. Exosomal miR-21 has been shown to induce M2 macrophage polarization via STAT3 signaling and suppress CD8+ T cell function (163). Notably, recent studies indicate that exosomal miR-21-5p also targets PDHA1 to promote glycolysis in glioblastoma, directly linking vesicle-mediated communication with metabolic reprogramming and the Warburg effect (164). Other molecules like TGF-β carried by EVs further amplify this immunosuppressive network by driving fibroblast activation.

## 10 Participation of non-coding RNAs in tumor immunity and transcriptome remodeling

### 10.1 The role of small non-coding RNAs in tumor immunity

In recent years, small non-coding RNAs (sncRNAs) have emerged as essential regulators of the complex interplay between tumor and immune cells. These RNAs, generally shorter than 200 nucleotides, include microRNAs (miRNAs), small interfering RNAs (siRNAs), PIWI-interacting RNAs (piRNAs), small nucleolar RNAs (snoRNAs), tRNA-derived small RNAs (tsRNAs), and small nuclear RNAs (snRNAs). Their functions extend across transcriptional, post-transcriptional, and epigenetic levels, thereby influencing both immune responses and tumor biology (165, 166).

sncRNAs exert critical regulation of immune and stroma cell functions during differentiation, proliferation, and effector activities. In particular, in immune cells sncRNAs critically shape the differentiation and activity of T cells, B cells, macrophages, and NK cells (167). miR-155 promotes Th1/Th17 polarization and supports M1 macrophage phenotypes, thereby enhancing antitumor immunity (168). In contrast, tumor-derived exosomal miRNAs such as miR-24-3p suppress T cell proliferation and facilitate immune escape (169). Likewise, snoRNAs such as SNORA38B have been implicated in promoting IL-10 secretion by lung cancer cells, which recruits Tregs and reinforces an immunosuppressive microenvironment (170). Beyond immune regulation, sncRNAs also contribute to remodeling the tumor transcriptome (171). Within tumor cells, they foster clonal heterogeneity, phenotypic plasticity, and therapy resistance. Evidence indicates that tsRNAs and piRNAs regulate critical signaling pathways such as JAK/STAT and PI3K/AKT/mTOR, supporting metabolic reprogramming and immune evasion (172). In addition, the role of several lncRNA regulating cancer-immunity cycle has been describe (97, 173).

Moreover, exosome-mediated release of sncRNAs functions as an intercellular communication system that reshapes the tumor microenvironment (174). By transferring regulatory cargo to immune or stromal cells, these vesicle-associated sncRNAs suppress CD8^+^ T cell cytotoxicity and reinforce tumor-promoting conditions (175).

During cancer treatment, a type of subpopulation of cancer cells emerges designated as drug-tolerant persistent (DTP) cells that survive to therapeutic options. DTP cells show limited proliferation, entering to a dormancy stage, metabolic changes that promote detoxification mechanisms, alterations in drug efflux systems, and non-genetic molecular modifications, such as epigenetic reprogramming, etc. Genetic and non-genetic modifications induce in DTP cells promote several signaling pathways associated with induction of immune evasion mechanisms and developing drug resistance. Although most studies have been conducted on clones of DTPs selected under pharmaceutical pressure, our group has reported transcriptomic changes in lung cancer cells exposed to a unique exposure to chemotherapeutic (cisplatin) or targeted therapy products (176, 177).

## 11 Regulating immune response in the tumor microenvironment

Deciphering the functional behavior of immune cells within the complex, dynamic TME is essential for optimizing strategies aimed at sustaining durable antitumor immunity. Advances in the characterization of TME composition have provided a clearer understanding of the mechanisms that allow tumors to evade antitumor immune activities (178). Notably, high macrophage infiltration often coincides with the presence of tumor-infiltrating lymphocytes (TILs), yet these T cells frequently display elevated expression of multiple inhibitory receptors that dampen their effector capacity.

This immunosuppressive landscape is shaped by the overexpression of immune checkpoint molecules that inhibit T cell activation and drive them toward functional exhaustion. Immune checkpoints encompass a diverse set of molecules with inhibitory or regulatory activity on T cells (179). Functionally, only glycosylated receptors are capable of delivering inhibitory signals, which they transmit through immunoreceptor tyrosine-based inhibitory motifs (ITIM) and immunoreceptor tyrosine-based switch motifs (ITSM) (180). While the intricate molecular mechanisms, receptor–ligand interactions and downstream signaling networks involved in their inhibitory effects are well documented elsewhere (179), they fall outside the scope of this section.

## 12 Combating cancer using the weapons of the immune system

The acquired immunity, particularly that of tumor-associated T cells due to their high capacity for clonal expansion in response to an antigen, is the predominant and novel immunotherapy against cancer. Nevertheless, other forms of cancer treatment are being applied with great success.

### 12.1 Preclinical therapeutic strategies

Preclinical efforts have increasingly demonstrated that effective modulation of the TME requires simultaneous intervention across stromal, metabolic, and immunological axes, moving beyond single-cell analysis to a spatially resolved understanding of multicellular niches (181). Among the most thoroughly investigated targets are CAFs, which orchestrate ECM remodeling, modulate immune infiltration, and create physical and biochemical barriers that limit therapeutic penetration. *In vivo* models have shown that selective depletion or functional reprogramming of CAF subsets—via fibroblast activation protein (FAP) inhibitors, CAF-specific CAR-T cells, or TGF-β–neutralizing traps—reduces collagen deposition, normalizes vessel perfusion, and enhances T cell infiltration. Importantly, scRNA-seq and spatial transcriptomics have refined this view, revealing that CAFs are not a uniform population (182). Distinct subtypes—such as LRRC15+ myofibroblastic CAFs (myCAFs) implicated in exclusion, versus IL-6-driven inflammatory CAFs (iCAFs)—exhibit divergent effects on tumor immunobiology (183). This supports the urgent need for precision targeting rather than global fibroblast elimination, aiming to revert reactive stroma to a quiescent, tumor-restrictive state.

Metabolic interventions constitute another major preclinical avenue, given the profound metabolic interdependence and competition between malignant cells and their stromal counterparts (184). Lactate export through MCT1/4 transporters maintains extracellular acidosis, which suppresses cytotoxic lymphocytes while promoting M2 macrophage polarization and stabilizing HIF-1α. Preclinical blockade of these transporters reduces lactate accumulation, restores T cell IFN-γ production, and sensitizes tumors to checkpoint inhibition. Similarly, the dependence of CAFs and macrophages on glutamine metabolism for collagen synthesis has enabled glutaminase inhibitors to attenuate protumorigenic cytokine networks. More recently, targeting lipid metabolism to induce ferroptosis—an iron-dependent form of cell death—has emerged as a potent strategy. Inhibiting the cystine/glutamate antiporter (system xc−) or GPX4 in the acidic TME can sensitize therapy-resistant mesenchymal cells to lipid peroxidation (185). Thus, targeting tumor–stroma metabolic symbiosis is evolving from simple nutrient deprivation to mechanistically exploiting metabolic vulnerabilities like oxidative stress and mitochondrial dynamics.

An area of growing interest is the modulation of EV signaling, which facilitates long-range communication across the TME and contributes to systemic immune dysregulation (186). Tumor-derived exosomes enriched with miR-21, TGF-β, or PD-L1 deliver suppressive cues that favor Treg expansion, inhibit NK-cell cytotoxicity, and blunt CD8^+^ T cell activation. Preclinical inhibitors of exosome biogenesis (e.g., nSMase2 inhibitors such as GW4869), Rab27-dependent release, or EV uptake have shown the capacity to disrupt these immunosuppressive circuits. Furthermore, emerging research highlights the role of the intratumoral microbiome, where bacterial-derived vesicles within the tumor niche can metabolize chemotherapeutics or sequester immune drugs, adding a new layer of complexity to EV-mediated resistance (187). Probably, EV-targeted therapies also reduce metastatic niche conditioning, highlighting their dual role in local and systemic tumor evolution.

Parallel advances in bioengineering have led to the development of nanoparticle-based intratumoral delivery systems designed to overcome the limited biodistribution of immunomodulatory agents (188). Nanoparticles functionalized with ligands for CAFs, TAMs, or endothelial receptors can concentrate immunostimulatory cargo—such as STING agonists, TLR ligands, IL-12, mRNA vaccines, or siRNA—directly within immune-excluded regions. A critical goal of these advanced platforms is not merely activation, but the induction of TLS—organized aggregates of immune cells within the tumor that predict superior immunotherapy response (189). Injectable hydrogels and bioresponsive scaffolds provide controlled cytokine release, enabling prolonged local immune activation without systemic toxicity. These platforms have demonstrated synergy with checkpoint inhibitors in murine models by reshaping the cytokine milieu, normalizing the tumor vasculature, and promoting the expansion of effector T cell populations.

Collectively, preclinical strategies now converge on a unified conceptual framework: the TME is not a passive bystander but an active, spatially organized therapeutic target whose reprogramming can unlock antitumor immunity. By dismantling stromal barriers, disrupting metabolic dependencies (including ferroptosis sensitivity), and intercepting vesicle-mediated immunosuppression, these interventions lay the groundwork for next-generation therapeutic combinations that aim to convert refractory, “cold” tumors into immunologically responsive and structured landscapes.

### 12.2 Clinical-stage interventions

Clinically validated strategies have evolved significantly beyond the foundational monotherapies targeting the PD-1/PD-L1 and CTLA-4 axes. The paradigm has shifted towards dual-checkpoint inhibition and the integration of novel modalities designed to bridge the gap between innate and adaptive immunity. A pivotal advancement in overcoming resistance is the FDA approval of LAG-3 (Lymphocyte-activation gene 3) blocking antibodies, such as relatlimab. Mechanistically, LAG-3 acts synergistically with PD-1 to enforce T cell exhaustion; thus, their combined blockade effectively reinvigorates exhausted CD8+ T cells in melanoma subsets that are refractory to single-agent therapy (190). Concurrently, the inhibition of TIGIT, another inhibitory receptor expressed on T cells and NK cells, is entering late-stage trials. TIGIT blockade prevents the binding of CD155 on tumor cells, thereby restoring the cytotoxic potential of NK cells and promoting a pro-inflammatory Th1 response, illustrating a move towards multi-targeted immune restoration (191).

Furthermore, the clinical landscape is being fundamentally reshaped by bispecific T cell engagers (BiTEs) and immune-mobilizing monoclonal TCRs (ImmTACs). These agents act as molecular bridges, physically linking T cells to tumor cells independently of the patient’s endogenous MHC-peptide presentation machinery. Notably, agents like tebentafusp—a gp100-peptide-HLA-directed CD3 T cell engager—have demonstrated that “cold” tumors with low mutational burdens, such as metastatic uveal melanoma, can be successfully targeted. By mechanically redirecting polyclonal T cells to the tumor site, tebentafusp bypasses the need for traditional antigen priming, turning an immunologically quiescent environment into an active inflammatory site (192).

Beyond pure immunotherapy, Antibody-Drug Conjugates (ADCs) have emerged as potent “Trojan horses” capable of remodeling the TME. These constructs deliver highly potent cytotoxic payloads (e.g., topoisomerase I inhibitors or auristatins) directly to cells expressing targets like HER2, TROP2, or Nectin-4. Crucially, next-generation ADCs utilizing cleavable linkers possess a significant “bystander effect”: after the payload is released within the target cell, it can diffuse across membranes to kill neighboring antigen-negative tumor cells and stromal fibroblasts (193). This mechanism not only induces direct tumor lysis but also triggers immunogenic cell death, releasing DAMPs and neoantigens that secondarily prime the host immune system, effectively converting the tumor into an *in situ* vaccine.

Concurrently, the success of mRNA technology has accelerated the development of personalized neoantigen cancer vaccines. Unlike non-specific immunostimulants, these vaccines rely on genomic sequencing of the patient’s tumor to identify unique mutations. Early-phase trials (e.g., mRNA-4157 combined with pembrolizumab) indicate that these vaccines can train the immune system to recognize patient-specific tumor neoantigens, promoting the expansion of high-avidity T cell clones that traffic to the TME, thereby reducing the risk of recurrence and demonstrating the feasibility of fully personalized immunotherapy (194).

Combination regimens are also becoming increasingly sophisticated, incorporating agents that modify the metabolic and physical barriers of the TME to overcome “immune exclusion.” Clinical trials are currently evaluating the blockade of the adenosinergic pathway (CD39/CD73/A2AR axis). High concentrations of extracellular adenosine in the TME, generated by the enzymatic degradation of ATP by CD39 and CD73, potently suppress T cell activity; inhibiting these enzymes prevents this metabolic suppression (195). Similarly, inhibitors of the VEGF/VEGFR pathway are being repurposed not just for anti-angiogenesis, but for vascular normalization. By correcting chaotic tumor vasculature, these agents reduce interstitial fluid pressure and upregulate adhesion molecules (e.g., VCAM-1) on endothelial cells, facilitating the extravasation and infiltration of effector T cells.

Finally, efforts to modulate the gut-tumor axis via Fecal Microbiota Transplantation (FMT) are advancing from preclinical concepts to clinical reality. Trials have shown that altering the gut microbiome—specifically enriching for commensals like Akkermansia muciniphila or Ruminococcaceae—can overcome resistance to anti-PD-1 therapy in refractory melanoma patients. This systemic modulation presumably works via metabolite signaling (e.g., short-chain fatty acids) and molecular mimicry, enhancing the baseline activation state of the immune system (196). Collectively, these advances mark a transition from broad immunosuppression reversal to a precise, mechanism-driven orchestration of the host-tumor interaction.

### 12.3 CAR-T cell therapy

Chimeric antigen receptor (CAR)-T cell therapy involves isolating T cells from allogenic or patient’s peripheral blood, genetically modifying them to express a synthetic receptor that recognizes unprocessed tumor-associated antigens, and expanding them *ex vivo* before reinfusion. Structurally, CAR-T cells contain an ectodomain with a single-chain variable fragment for antigen recognition, a transmembrane domain, and an intracellular region harboring one or more signaling domains. Based on the number of intracellular signaling modules, CAR-T cells are categorized into different generations (197).

Upon antigen engagement, CAR-T cells release cytokines or express co-stimulatory molecules that establish a proinflammatory milieu, recruiting and activating other immune effectors to eliminate malignant cells. While CAR-T therapy has transformed the treatment of hematologic malignancies, its success in solid tumors has been modest, limited by the immunosuppressive TME and physical stromal barriers (198). Overcoming these obstacles is now a significant research focus.

Beyond lymphoid lineage, CAR-myeloid cells (such as CAR-macrophages) represent a novel frontier. Unlike T cells, CAR-macrophages can actively infiltrate dense solid tumors and remodel the immunosuppressive TME, offering a potential solution to the physical and metabolic barriers that limit current CAR-T therapies (199).

To overcome the limitations of traditional CAR-T therapies in solid tumors, such as physical barriers and immunosuppressive TME, recent advances have focused on diversifying the cellular platforms. CAR-NK cells have emerged as a promising ‘off-the-shelf’ alternative, offering potent antitumor activity with a reduced risk of cytokine release syndrome and graft-versus-host disease compared to T cells. Furthermore, CAR-myeloid cells (CAR-M) are being engineered to exploit their innate ability to infiltrate dense tumor stroma and phagocytose malignant cells, effectively turning ‘cold’ tumors ‘hot’ by remodeling the local immune microenvironment. Innovations such as induced pluripotent stem cell (iPSC)-derived cell products and multifunctional CAR designs are currently being optimized to enhance persistence and prevent antigen escape (200).

It is imperative to distinguish between established clinical interventions and emerging preclinical strategies. Clinically, immune checkpoint inhibitors (ICIs) and CAR-T therapies have reshaped the landscape for hematologic and some solid tumors, yet they face hurdles regarding durability and resistance (104). In contrast, the preclinical frontier is dominated by bioengineering approaches aimed at overcoming these limitations. For instance, recent studies have demonstrated the efficacy of engineered EVs derived from M2 macrophages or functionalized nanoparticles to deliver immunomodulatory cargo directly to the TME (201, 202). Furthermore, functionalized inorganic nanomaterials are being optimized for precise drug delivery and tracking (203), representing a promising ‘next-generation’ tool not yet available in routine practice (204).

### 12.4 Immune checkpoint inhibitor therapy

Immune and stromal cells naturally express immunomodulatory molecules that safeguard self-tolerance and prevent autoimmunity. Tumor cells hijack this regulatory system by overexpressing ligands for inhibitory receptors, collectively known as immune checkpoints, to suppress T cell activation and evade immune attack. The development of therapeutic antibodies targeting these checkpoints has revolutionized solid tumor treatment. By blocking ligand–receptor interactions, ICIs prevent inhibitory signaling cascades, allowing antigen-experienced T cells within the TME to regain their cytotoxic functions. Additional efforts have been made with the recent development of CAR-NK cells. Whether as monotherapy to triple combination or in rational combinations of several other therapeutic options, ICIs continue to reshape the therapeutic landscape in oncology (178, 205).

### 12.5 Endogenous EVs engineering

The capture and identification of EVs at the serum level using liquid biopsy has been proposed as suitable biomarkers for cancer detection, its progression, and treatment monitoring. Research confirms that the release of EVs contents reprograms the phenotype and functionality of target cells, which has become an opportunity for therapeutic applications. Based on engineering strategies, it has been proposed to load various types of biological molecules into EVs. The supply of immunogenic antigens to increase the reactivity of immune cells against cancer, or the use of vectors that inhibit the production of pro-tumor cytokines, promote the participation of transcription factors related to the improvement of anti-tumor activities or the blocking of pro-tumor activities, to the loading of natural or synthetic therapeutic biomolecules, are some of the current challenges. To this aim, different methodological procedures are being tested for loading into EVs, transporting the load to specific cells, and its fusion with cell membranes.

### 12.6 Other strategies for cancer treatments

These include the production of cytokines with pro- or anti-inflammatory activities using different technological formats and their application to patients, the adoptive transfer of tumor-infiltrating lymphocytes (TILs) obtained directly from tumor tissue, lymphocyte-activated killer cells (LAK cells) from peripheral blood cells activated *in vitro* with IL-2, as pioneering treatments for cancer, and DCs therapy, consisting in differentiate immature DCs to mature phenotype, stimulating them *in vitro* with pool of cytokines and tumor antigen, NK cell therapy including diverse options, the development of vaccines against neoantigens or novel targets, until the developing T cell engineering using CRISPR-Cas9 or base editors. Recent publications describing the varied development of different cancer therapies have been recently published (206–209).

Taken together, these insights suggest that the most effective therapeutic strategies will be those capable of disrupting the communication networks within the tumor microenvironment, rather than targeting isolated cell types. A coherent approach involves simultaneously restoring effector immune function and neutralizing dominant suppressive circuits, for example by combining ICIs with inhibitors of adenosine signaling (CD39/CD73/A2A) or with modulators of the IDO/TDO–kynurenine–AhR pathway, and by integrating TGF-β antagonists to overcome stromal exclusion (210). In parallel, reprogramming the stroma, through redirection of TAMs via CSF1R or PI3K-γ blockade and by targeting key CAF functions using FAP-directed agents, TGF-β traps, or ECM-normalizing approaches, can enhance perfusion, drug delivery, and lymphocyte infiltration (211). Because metabolic cooperation underlies resistance and immune dysfunction, strategies that interrupt metabolic crosstalk, such as inhibition of lactate shuttling (MCT1/4), buffering of acidosis, or suppression of hypoxia/HIF signaling, may further weaken immune evasion and EMT (212). Additional opportunities arise from limiting vesicle-mediated communication and platelet assistance, aiming to reduce exosome biogenesis or uptake and mitigate platelet-driven protection and angiogenesis. Finally, the next generation of immunotherapies focuses on engineering resilient immune effectors, including armored CAR-T/CAR-NK cells, bispecific engagers, and oncolytic/immunogenic platforms designed to resist TGF-β, adenosine, and nutrient scarcity, capable of maintaining activity despite the profound suppressive pressures of the TME (213).

Furthermore, recent advances in nanotechnology and functionalized nanomaterials mimic cellular communication mechanisms to deliver therapeutic payload specifically to the TME, overcoming biological barriers (176).

### 12.7 Resistance perspectives

Therapeutic resistance emerges from a complex interplay between tumor-intrinsic alterations and extrinsic adaptations governed by the TME. While intrinsic resistance relies on pre-existing genomic and epigenomic aberrations—such as *TP53* mutations or alterations in drug target—clinical evidence suggests that these factors alone are insufficient to explain therapeutic failure. Instead, extrinsic resistance, often termed “environment-mediated drug resistance”, arises from reciprocal crosstalk between tumor cells, stromal components, and infiltrating immune cells. This interaction fosters a protective niche that ensures survival under cytotoxic pressure through four distinct mechanisms: physical barrier formation, pro-survival signaling, metabolic reprogramming, and immune exclusion.

CAFs actively remodel the ECM by depositing dense collagen, fibronectin, and hyaluronan. This desmoplastic reaction increases tissue stiffness and significantly elevates interstitial fluid pressure (IFP). High IFP compromises vascular integrity, leading to vessel collapse and impaired perfusion. Crucially, this disrupts the hydrostatic pressure gradient necessary for the transvascular diffusion of therapeutics, causing drugs to accumulate in the periphery while failing to penetrate the tumor core (214, 215). This biophysical barrier severely limits the efficacy of both small-molecule cytotoxic agents and larger monoclonal antibodies.

However, the implementation of multi-targeted strategies faces acute challenges, primarily synergistic toxicity and adaptive resistance. Off-target toxicity: Systemic immune activation can trigger severe immune-related adverse events (irAEs). Recent reports highlight that unchecked immune responses can manifest as life-threatening conditions, such as hemophagocytic lymphohistiocytosis (HLH) with neurological involvement, mimicking autoimmune demyelinating disorders (216). These severe toxicities often force dose reductions that compromise the antitumoral efficacy of combinatorial regimens.

Spatial Heterogeneity: Therapeutic failure is also driven by physical and biological barriers. High preoperative fibrinogen levels, for example, have been identified as a prognostic marker associated with poor survival and recurrence, indicative of a dense, pro-tumorigenic ECM that hampers drug penetration (217). Additionally, macrophage plasticity contributes to this exclusion; TAMs can dynamically shift phenotypes via exosomal miRNA exchange, creating immunosuppressive niches that are impenetrable to standard T-cell therapies (218). Furthermore, emerging mechanisms such as chemoresistance driven by specific TME interactions continue to limit the efficacy of standard regimens (147).

Beyond structural impediments, the TME functions as a signaling hub that antagonizes therapy-induced apoptosis. CAFs and TAMs secrete a plethora of cytokines and growth factors, including IL-6, HGF, and IGF-1. These ligands activate critical downstream pathways in tumor cells, such as PI3K/AKT, JAK/STAT3, and MEK/ERK (182, 219). The activation of these cascades inhibits apoptotic machinery, induces epithelial–mesenchymal transition (EMT), and promotes a “stem-like” phenotype associated with drug-tolerant states. Furthermore, Tregs and myeloid-derived suppressor cells (MDSCs) reinforce this resistant ecosystem by secreting immunosuppressive cytokines (IL-10) that impair effector T cell priming. Furthermore, long non-coding RNAs such as *Lnc-TMEM132D-AS1* have been implicated in acquired resistance to targeted therapies like osimertinib, representing a new layer of regulatory complexity (171).

Finally, the TME orchestrates resistance through spatial exclusion and horizontal transfer of resistance traits. A rich stroma and dense collagen networks physically exclude cytotoxic T cells from tumor islets. These T cells become trapped in the peritumoral stroma, resulting in an “immune-excluded” phenotype that responds poorly to ICIs (220). Additionally, resistance is propagated horizontally via EVs (186).

## 13 Conclusion and perspectives

**Figure 3:**
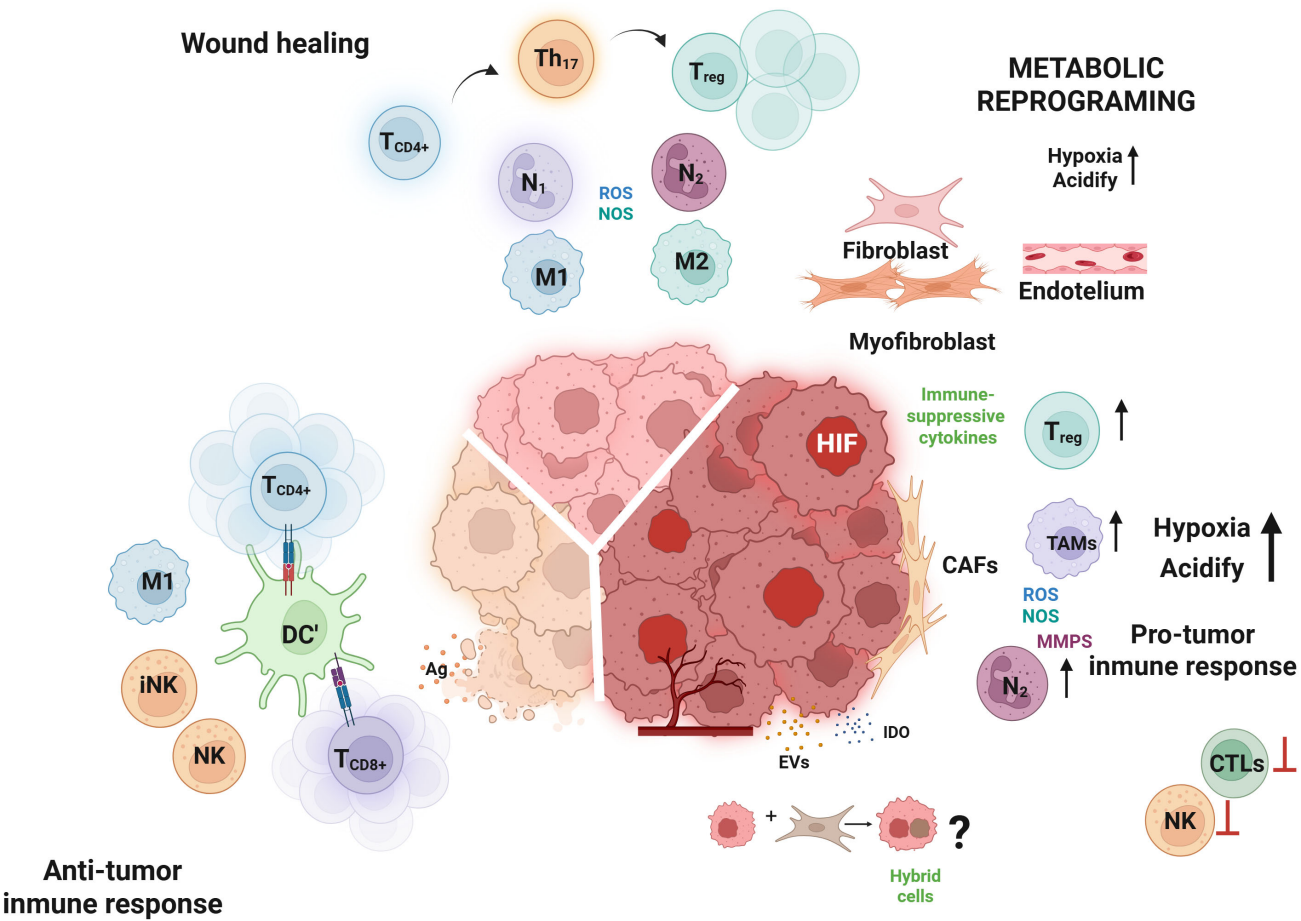
A simplified overview of the interaction between different cells throughout tumor development. It highlights the participation of innate and adaptive immune cells that act against local transformed cells and their gradual adaptation to the tumor environment, where they acquire pro-tumor activity, as well as the participation of stromal cells and components. It also includes events such as oxidative stress, metabolic reprogramming, the involvement of extracellular vesicles, and the possible generation of hybrid cells. All these factors can trigger the development of various resistance mechanisms by the tumor to immune response and promote angiogenesis, which are involved in cancer progression.

In this review, we depart from the evolutionary oncology approach and its close relationship with the different immune and stromal cells that characterize the tumor microenvironment. Based on excellent reviews detailing new technologies and procedures, we highlight the different mechanisms of cellular communication with the aim of presenting a multifaceted and comprehensive point of view. This review has outlined how multidirectional crosstalk between tumor, immune, and stromal cells, via soluble mediators, membrane ligands, extracellular vesicles, and direct physical interactions, reprograms tissue physiology. This network modulates immunoediting, drives metabolic reconfiguration, involves tissue repair cells and molecules, promotes invasion and angiogenesis, enables immune evasion, and ultimately leads to tumor resistance (See **Figure 3**).

We highlight the role that tissue repair plays in promoting tissue conditions that may act as potential triggers for tumor development. In addition, central regulatory nodes included canonical signaling cascades (JAK/STAT, PI3K/AKT/mTOR, TGF-β, cadherin-catenin/WNT), metabolic and redox programs (HIF-1, lactate, ROS), immunoregulatory circuits (IDO/TDO-kynurenine-AhR), adenosine via CD39/CD73), and key microenvironmental players such as TAMs, CAFs, platelets, and TLS, EMT, and cell-cell fusion add greater tumor plasticity by increasing heterogeneity and resistance to treatment.

Progression from immune defense to tumor promotion is not a single binary switch but a network phenomenon. Nutrient depletion (e.g., arginine), acid–hypoxia gradients, and vesicle trafficking act in concert with checkpoint signaling to blunt cytotoxic responses and polarize myeloid and fibroblast phenotypes toward tumor support. This interconnectedness might explain why monotherapies often fail in solid tumors: disabling one pathway leaves much of the circuit intact (81).

Advancing the field will require mapping tumor–stroma–immune communication with spatial, temporal, and causal precision. Spatial multi-omics and intravital imaging must be integrated with functional perturbation screens to decipher intercellular crosstalk—revealing precisely which cells interact, when, and with what consequence. Longitudinal sampling, utilizing liquid biopsies to analyze vesicle cargo and soluble mediators, offers a means to track TME dynamics throughout the course of therapy. Furthermore, functional preclinical models, such as patient-derived organoids and ex vivo immune–stroma co-cultures, are essential for testing circuit-level interventions under physiologically relevant conditions of hypoxia, acidity, and matrix complexity. The biology of cell fusion and hybrid states warrants dedicated investigation, as does the standardization of TLS quantification and maturation scoring. Consequently, communication biomarkers should be developed to guide combination therapies, ideally incorporating pharmacodynamic readouts that capture TME rewiring in real time.

Finally, the immense complexity of tumor heterogeneity and antigen escape necessitates precision that transcends conventional methodologies. The integration of artificial intelligence (AI) and machine learning has become indispensable for the next generation of immunotherapies. Emerging tools, such as AI-assisted antigen screening and computational modeling, are now being leveraged to predict optimal targets and design combinatorial regimens capable of effectively rewiring TME communication networks, thereby tailoring treatments to the unique molecular landscape of each patient.

In summary, decoding and deliberately reprogramming the languages of cellular crosstalk may transform oncology from targeting isolated nodes to rewiring entire tumor ecosystems. Done effectively, the TME can be converted from cancer’s accomplice into its Achilles’ heel, turning hostile microenvironments into therapeutic opportunities. This review is not intended as an exhaustive mechanistic analysis but rather as a comprehensive reference framework that synthesizes key concepts and literature for researchers investigating tumor–stroma–immune interactions.
